# Real-Time Dosimetry in Endourology: Tracking Staff Radiation Risks

**DOI:** 10.3390/diagnostics14161763

**Published:** 2024-08-13

**Authors:** Susanne Deininger, Olaf Nairz, Anna Maria Dieplinger, Christian Deininger, Lukas Lusuardi, Christian Ramesmayer, Julia Peters, David Oswald, Maximilian Pallauf, Sophina Bauer, Mathias Christoph Brandt, Peter Törzsök

**Affiliations:** 1 Department of Urology and Andrology, Salzburg University Hospital, Paracelsus Medical University, 5020 Salzburg, Austria; l.lusuardi@salk.at (L.L.); c.ramesmayer@salk.at (C.R.); d.oswald@salk.at (D.O.); m.pallauf@salk.at (M.P.); s.bauer@salk.at (S.B.); torzsok.peter@gmail.com (P.T.); 2 Radiation Protection Office, Salzburg University Hospital, Paracelsus Medical University, 5020 Salzburg, Austria; o.nairz@salk.at; 3 Institute for Nursing Science and Practice, Paracelsus Medical University, 5020 Salzburg, Austria; annamaria.dieplinger@ooeg.at; 4 Institute of Tendon and Bone Regeneration, Paracelsus Medical University, 5020 Salzburg, Austria; 5 Department of Orthopedics and Traumatology, Salzburg University Hospital, Paracelsus Medical University, 5020 Salzburg, Austria; 6 Department of Cardiology, Salzburg University Hospital, Paracelsus Medical University, 5020 Salzburg, Austria; m.brandt@salk.at; 7 Faculty of Health and Sport Sciences, Széchenyi István University, 9026 Győr, Hungary

**Keywords:** endourology, X-ray, radiation protection, staff, stone removal, lead apron, eye lens, RIRS

## Abstract

Background: To retrospectively investigate scatter radiation (SCR) exposure among staff in the endourology operating theatre. Methods: During surgeries under fluoroscopic guidance, five professional groups (urological surgeon [US], surgical nurse [SN], assistant surgical nurse [ASN], anaesthetist [A], and anaesthesia care [AC]) wore real-time dosimeters (Philips DoseAware System) on their head and chest over lead aprons between July 2023 and February 2024. The SCR data were analysed and correlated with procedural and patient factors. Results: In total, 249 procedures were performed, including 86 retrograde intrarenal surgeries and 10 percutaneous nephrolithotomies. Median SCR exposure was 38.81, 17.20, 7.71, 11.58, 0.63, 0.23, 0.12, and 0.15 Microsievert (µSv) for US chest (USC), US head (USH), SN chest (SNC), SN head (SNH), A chest (AC), AC chest (ACC), ASN chest (ASNC), and ASN head (ASNH), respectively. There was a significant correlation between DAP and SCR doses detected by USC, USH, SNC, SNH, AC, and ACC dosimeters (*p* < 0.05). The median chest-to-eye conversion factor (CECF) was 2.11 for the US and 0.71 for the SN. Conclusions: This study, using real-time dosimetry, is among the first to assess staff occupational SCR exposure in endourology. It highlights a substantial SCR exposure, indicating an occupational health hazard that warrants further investigation.

## 1. Introduction

Since the discovery of X-ray radiation by Wilhelm Conrad Röntgen in 1895, X-rays have increasingly been established as an important tool in the field of urology. As early as 1926, Eugen Joseph noted that the collection of all relevant urological X-ray images in an atlas would be helpful and that “knowledge of the normal silhouette of the urinary tract [would be] absolutely necessary for the comparison with the pathological shape” [1,2].

Currently, X-ray imaging is being implemented in urological departments worldwide. Procedures for stone management (SM), such as retrograde intrarenal surgery (RIRS), percutaneous nephrolithotomy (PCNL), and the placement or change of ureteral stents require the use of X-ray imaging for visualisation and orientation within the urinary tract. Gul et al. (2023) published a data analysis of urological departments across all American medical colleges. In their study, RIRS with laser lithotripsy was identified as one of the five most commonly performed urological procedures [3]. Silvestre et al. (2020) also described endourology/SM as one of the most common urological surgical procedures, with 21–24% of surgeries performed by graduating urology residents [4]. Furthermore, the incidence of urolithiasis is rising: According to an analysis of the Global Burden of Disease study, the incidence increased in all regions during the period of 1990–2019, except for Eastern Europe, Central Europe, and Southeast Asia [5]. SM (RIRS, PCNL) and many other endourological procedures involve the use of X-rays. 

The use of ionising radiation in medicine is discussed controversially. While on the one hand being the driver for more and more sophisticated treatment techniques, ionising radiation can have detrimental health effects for both patients and the healthcare staff exposed. A solid research basis has been established quantifying patient radiation exposure via the “central beam” area during fluoroscopic surgeries. However, little is known about the level of chronic exposure to the so-called “scattered radiation” (SCR) that the operating team is frequently affected by in the endourology operating theatre. Chou et al. (2015) conducted a questionnaire survey among 328 American female urologists, and they found that 54.3% of the participants reported performing fluoroscopy at least once a week, while 37.5% performed fluoroscopy at least once a month [6]. The fact that chronic exposure to SCR has serious health effects has been well established in other medical professions.

The aim of this study was to evaluate the SCR exposure of various professional groups working in the endourology operating theatre and to correlate it with the dose–area product (DAP), numerous clinical factors of the patients and the surgeries performed, as well as with the professional experience in the US. 

## 2. Material and Methods

### 2.1. Data Recording and Analysis 

Data on patient characteristics, procedural details, fluoroscopy data, and SCR doses measured for participating staff groups were recorded and analysed as an amendment of the prospective “Occupational SCAtter Radiation Registry” (OSCAR Registry). The study was approved by the Ethics Committee of the Province of Salzburg, Austria, (Reference number 1069/2021, approval date 9 June 2021) and listed on clinicaltrials.gov (Identifier #NCT04945538). Data analysis was conducted as a collaboration between the University Clinic for Urology and Andrology, Salzburg, Austria, and the Radiation Protection Office of the State Hospital, Salzburg, Austria. This is a retrospective analysis of data, which were recorded between 26 July 2023 and 21 February 2024. 

Knee-length lead aprons and thyroid shields (lead equivalent thickness 0.5 mmPb) from various manufacturers were worn by the entire staff as standard in the endourology operating theatre. Lead-glass spectacles were not worn regularly. Passive personal dosimeters were worn on the chest underneath the lead apron as required by legal regulations. During the study period, eight real-time dosimeters (Philips DoseAware System, Philips Medical Systems Nederland B.V., Veenpluis 6, 5684 PC Best, The Netherlands) were additionally worn by five different professional groups, urological surgeon (US, head and chest), surgical nurse (SN, head and chest), assistant surgical nurse (ASN, head and chest), anaesthetist (A, chest), and anaesthesia care (AC) during various endourological procedures. 

**Figure 1:**
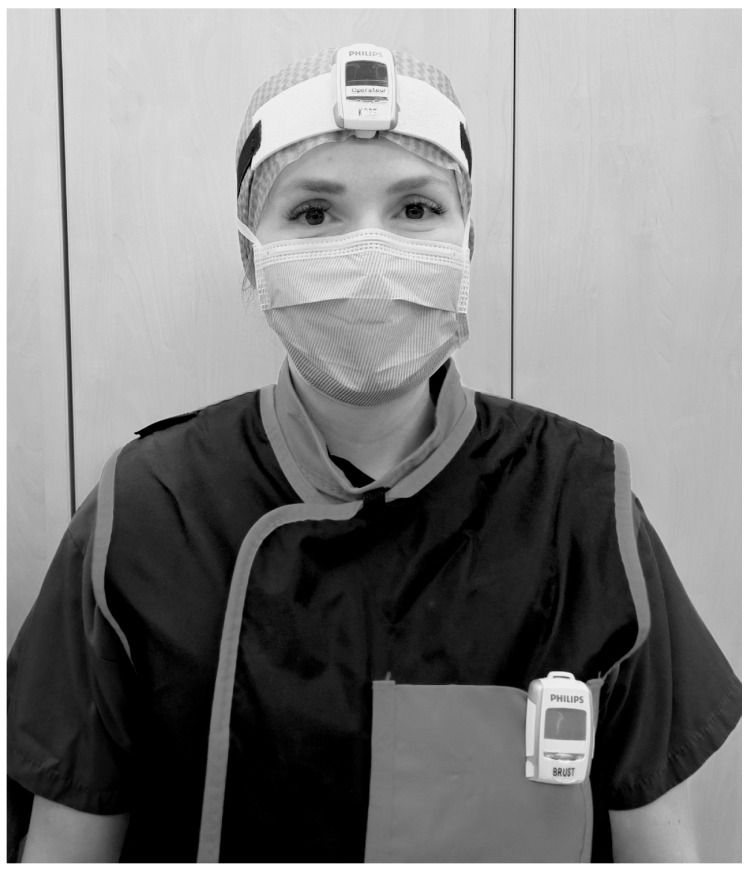
Method of wearing of the real-time dosimeters on the head (between the eyes on the forehead attached to a headband or the surgical cap) and chest (on the left chest above the X-ray apron).

The dosimeters were worn above the lead apron on the left chest and between the eyes on the forehead attached to the surgical cap or attached to a band (see Figure 1). The dosimeters were assigned only to the professional group and the body part, not to specific individuals. 

**Figure 2:**
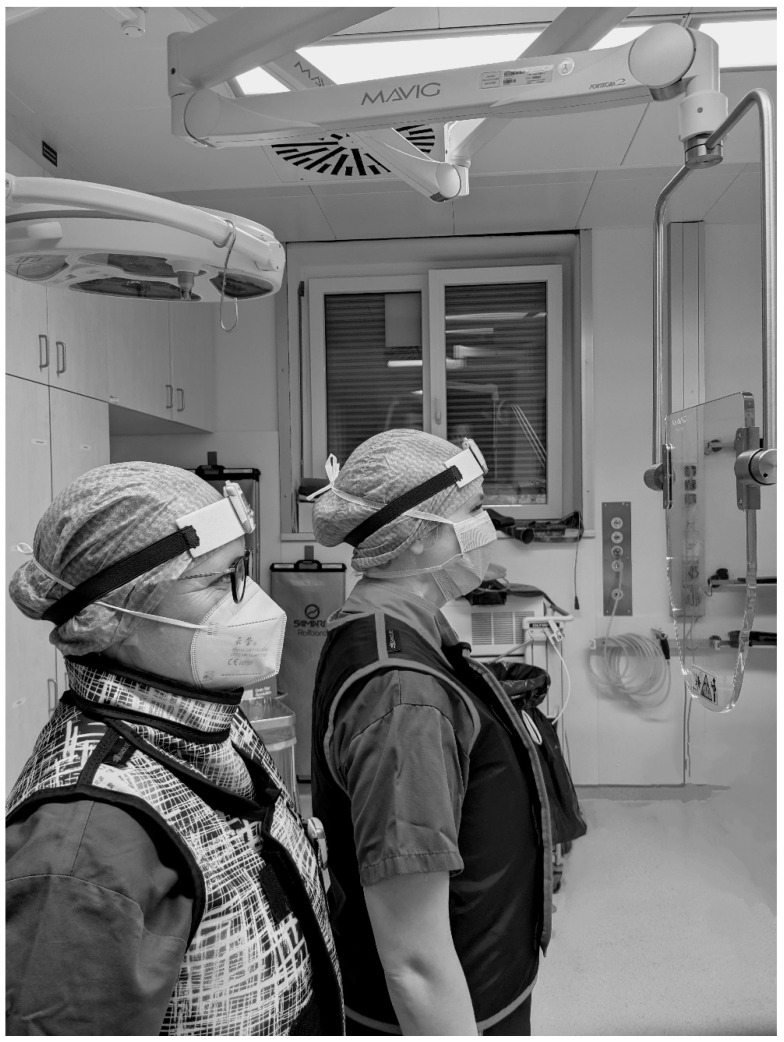
Illustration of the positioning of the radiation protective shield “OT81001” (MAVIG GmbH, Munich, Germany) in the area between patient and urological surgeon (right) and surgical nurse (left).

In some cases, the additional radiation protective shield “OT81001” (MAVIG GmbH, Postfach 82 03 62, 81803 München, Germany; lead equivalent thickness 0.50 mmPb) was implemented (see Figure 2). 

The corresponding dose area products (DAPs) were retrieved from the dose management system (EasyDose^QM^, BMS Informationstechnologie^®^ Gesellschaft m.b.H, Vienna, Austria). Individual SCR recordings from the dosimeters were assigned to the corresponding procedures using automatic dose reports and electronic surgical reports.

The X-ray system used is integrated in the multifunctional urological workstation MODULITH SLX-F2 »connect« (STORZ MEDICAL AG, Lohstampfestrasse 8, 8274 Tägerwilen, Switzerland), with a voltage range between 40 kV and 125 kV for fluoroscopy and between 40 kV and 150 kV for radiography. The US operated the X-ray using a foot pedal during the procedures.

The type, date, and duration of the procedure, as well as the side involved, were recorded. 

**Figure 3:**
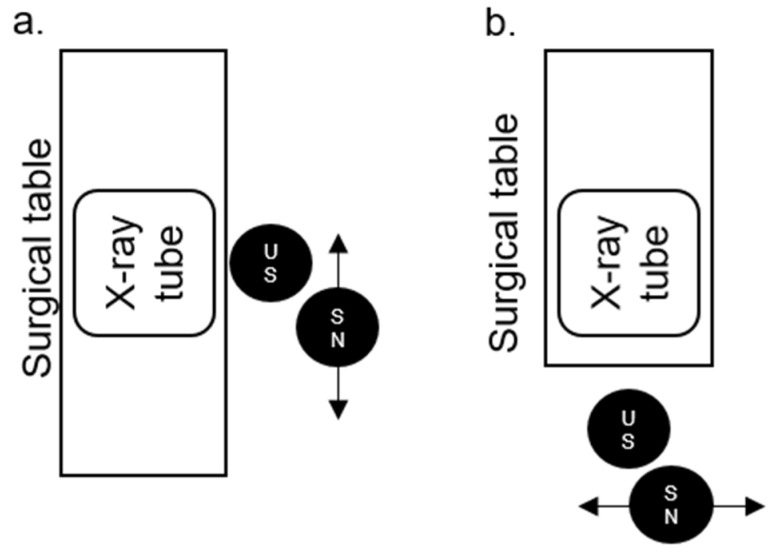
The positions of the urological surgeon (US) and surgical nurse (SN) in relation to the surgical table, X-ray tube, and to each other in the percutaneous nephrolithotomy (PCNL) (**a**) and lithotomy position (**b**).

The positions of the US and SN in relation to the surgical table, X-ray tube, and to each other in the PCNL position (Figure 3a, for PCNL, nephrostomy and MJ ureteral stents) and lithotomy position (Figure 3b, for all other mentioned procedures) are illustrated below.

If retrograde intrarenal surgery (RIRS) or PCNL was performed, the stone size, position, chemical composition of the stone, and postoperative stone-free status (SFS) were also recorded. Additionally, various clinical data were analysed, including the patient’s age, sex, weight, height, BMI, and the use of anticoagulants or antiplatelet drugs. 

Participating urological surgeons were categorised into groups according to their level of expertise (resident physicians, urology specialists, and urology experts, including senior physicians and head of department). A comparison of the emitted radiation doses (i.e., DAP) and the recorded real-time dosimetry data was conducted based on the professional experience of the US. In order to provide time-series data, DAP and SCR data were recorded for 26 consecutive procedures performed by a designated resident assistant physician who had just started his career in the endourology operating theatre (learning curve analysis).

### 2.2. Statistics 

Continuous variables were analysed for normal distribution (Kolmogorov–Smirnov test with Lilliefors correction, type I error = 10%) and homoscedasticity (Levene test, type I error = 5%).

Comparisons between residents, specialists, and experts were conducted using parametric ANOVA for normally distributed data with equal variances (post hoc by Hochberg’s GT2 method) and non-parametric ANOVA (Kruskal–Wallis test followed by Nemenyi’s multiple comparisons) for other continuous variables. Categorical variables were compared using the chi-square test, exact or with Monte Carlo simulation, providing adjusted residuals.

For comparing dosimeter readings between the surgeon’s and the surgical nurse’s chest/head, the exact Wilcoxon test was used due to non-normal distribution of data.

Correlations of non-normally distributed continuous variables with continuous or dichotomous variables were assessed using the Spearman’s rank correlation coefficients. Relationships between continuous and categorical variables with more than two categories were represented by eta^2^ coefficients and Kruskal–Wallis test *p*-values.

Multiple linear regression analyses examined variables influencing the dosimeter readings (independent variables: surgeon, age, BMI, operating time, sex, radiation protective shield use, and urinary stone analysis) and the DAP for surgeon 1 (independent variables: age, BMI, operating time, OP-sequence surgeon 1, sex). Regression lines had a zero intercept for dosimeter variables as dependent and the dose area product as independent variables.

No adjustment was made for multiple testing, making the inferential statistics results descriptive. Analyses were performed using R version 4.3.1 (The R Foundation for Statistical Computing, Vienna, Austria).

## 3. Results

During the study period, *N* = 249 procedures were performed using fluoroscopy, with at least one of the dosimeters in place during the entire surgery. Key characteristics of the patients and surgeries performed are listed in Table 1.

### 3.1. Dose Area Product

**Figure 4:**
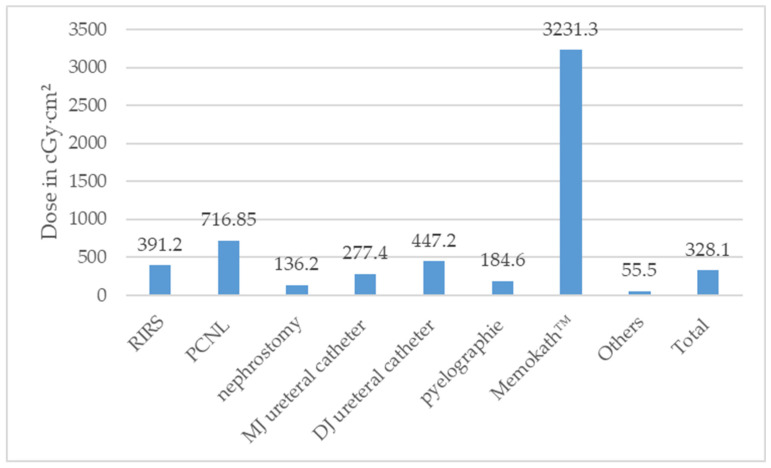
Median dose area product (DAP) in Centigray (cGy)·cm^2^ according to type of surgeries performed (ESWL = extracorporeal shock wave lithotripsy, PCNL = percutaneous nephrolithotomy, RIRS = retrograde intrarenal surgery).

According to the type of surgery, the median DAP stratified is illustrated in Figure 4 and Table S1. 

### 3.2. Correlation of DAP with Clinical Factors 

A statistically significant correlation was found between the dose area product (DAP) and the patient’s age (** *p* < 0.001), BMI (** *p* < 0.001), weight (** *p* < 0.001), and the duration of the surgery (** *p* < 0.001). There was no correlation between DAP and the patient’s height (*p* = 0.37), maximum stone size in RIRS (*p* = 0.10), the patients’ gender (*p* = 0.88), indication for RIRS (*p* = 0.08), SFR (*p* = 0.36), or the patients’ use of anticoagulants or antiplatelet medication (*p* = 0.59)

### 3.3. Correlation of DAP and Clinical Factors with the Professional Experience of the US 

The number of surgeries performed by the US, with their respective level of experience, can be found in Table S2. As patients were stratified according to the corresponding group of physicians performing the surgeries, there were no significant differences in the demographic patient data (BMI (*p* = 0.16), sex (*p* = 0.05), the use of anticoagulants or antiplatelet therapy (*p* = 0.73)), procedure-specific data (for RIRS maximum stone size (*p* = 0.68), indication (*p* = 0.78), the side (*p* = 0.14), the use of laser (*p* = 0.32), SFR (*p* = 0.60), urinary stone analysis (*p* = 0.24), and the stone location (*p* = 0.96)). Differences were observed, however, between various levels of professional expertise and patient age (** *p* < 0.001), DAP (* *p* = 0.04), duration of the surgery (** *p* < 0.001), and the type of surgery (** *p* < 0.001).

### 3.4. Learning Curve Analysis of One Designated Resident Physician 

Assuming that the procedural duration and DAP should decrease with increasing surgical experience (i.e., surgery sequence), one designated resident physician in his second year of training, who had just begun his career in the endourology operating theatre, was evaluated longitudinally. During the time period, he performed 26 endourological procedures: *n* = 8 change or placement of nephrostomy, *n* = 13 change or placement of MJ ureteral catheter, and *n* = 5 change or placement of DJ ureteral catheter. However, no correlation was found between the DAP and the sequence of surgeries (*p* = 0.50). Therefore, no learning curve regarding the use of X-rays was observed for this US during the observation period.

### 3.5. Analysis of Individual Real-Time Dosimeters 

**Figure 5:**
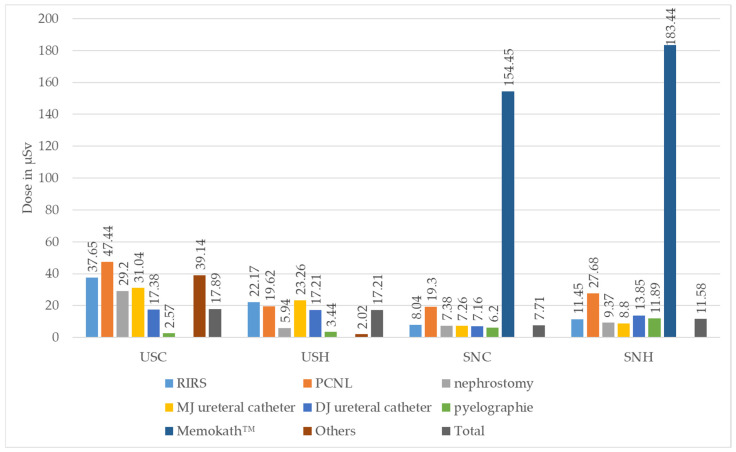
Median absolute values of individual real-time dosimeters in Microsievert (µSv) for urological surgeon chest (USC) and head (USH) and for surgical nurse chest (SNC) and head (SNH), stratified according to type of surgery performed (ESWL = extracorporeal shock wave lithotripsy, PCNL = percutaneous nephrolithotomy, RIRS = retrograde intrarenal surgery. Comment: Measurement for USH/Memokath™ removed due to implausibly low recording).

The median values of the individual real-time dosimeters analysed according to the type of surgery can be found in Table 2 and Figure 5. 

### 3.6. Correlation of DAP with Individual Real-Time Dosimeters

**Figure 6:**
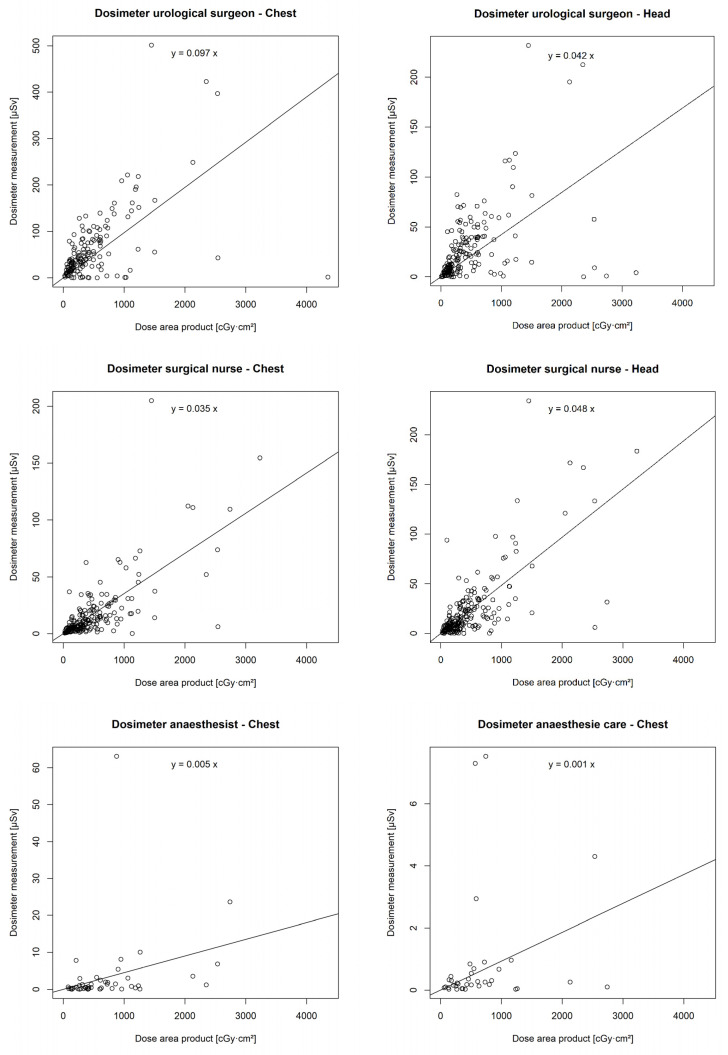

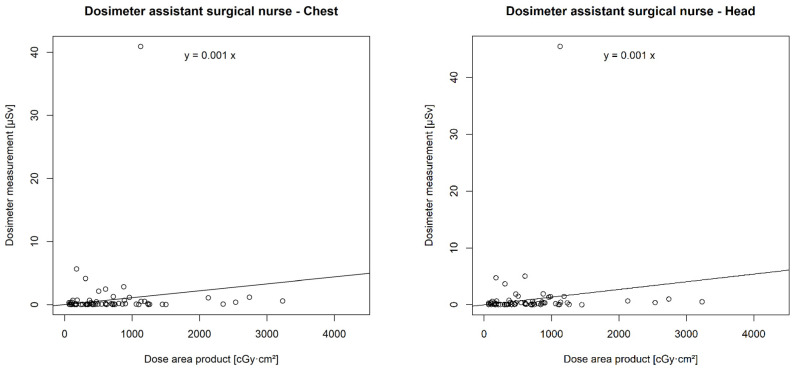
Correlation of individual real-time dosimeters’ values in µSievert (µSv) with dose area product (DAP) in centigray (cGy)·cm^2^.

There was a statistically significant correlation between the DAP and the chest (*p* < 0.001 **) and head dosimeters (*p* < 0.001 **) of the urological surgeon, the chest (*p* < 0.001 **) and head dosimeters (*p* < 0.001 **) of the surgical nurse, the chest dosimeter of the anaesthetist (*p* = 0.001 *), and the chest dosimeter of the anaesthesia care (*p* = 0.047 *). However, no correlation was found between the DAP and the chest (*p* = 0.14) or head dosimeter (*p* = 0.12) of the assistant surgical nurse (Figure 6). 

### 3.7. Influence of the Use of an Additional Lead Shield on Individual Radiation Exposure 

In six cases, a ceiling-supported lead shield (CSLS) at the level of the surgeon’s head was used as an additional radiation protection measure (see Figure 2): five cases of RIRS (two stone removals, three diagnostic/oncological) and one case of change or placement of DJ ureteral catheter. 

**Figure 7:**
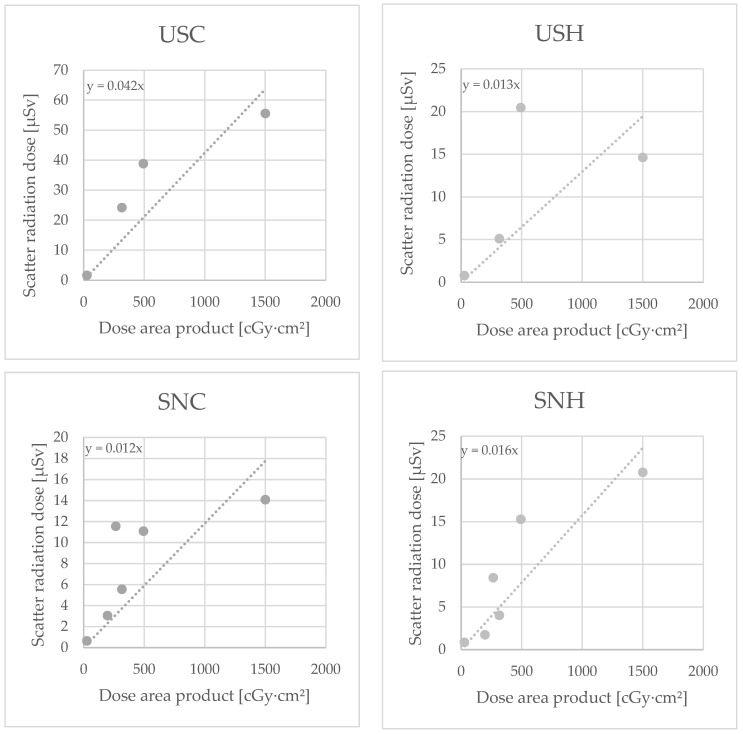
Dosimeter measurements with use of additional lead shield for the dosimeters urological surgeon chest (USC) and head (USH) and surgical nurse chest (SNC) and head (SNH), in Microsievert (µSv)/Centigray (cGy)·cm².

Subgroup analysis of these procedures (Figure 7) showed a trend towards SCR dose reduction. This trend, however, was not statistically significant. The use of a CSLS had no effect on SCR exposure on the chest (*p* = 0.479) or the head of the surgeon (*p* = 0.127), or on the chest (*p* = 0.274) or the head of the surgical nurse (*p* = 0.203), compared with surgeries without the use of a lead shield. 

### 3.8. Chest-to-Eye Conversion Factor (CECF) 

In each case where SCR recordings for both the chest and the head dosimeters of the US and the SN were available, a correlation analysis between the two values was performed (CECF = chest-to-eye conversion factor). The results are illustrated in Table 3.

This shows that the US seems to have more exposure in the chest area, while the SN has more exposure in the head area (i.e., eye lens).

## 4. Discussion 

Today, operative urology, in particular endourology, is unthinkable without the application of X-rays; yet, its use remains controversial. X-rays are ionising radiation, which means that they can cause permanent damage to human cells. This is particularly relevant with regular exposure in a work environment involving radiation. 

X-ray exposure can induce malignant diseases [7] in healthcare workers. A review by Gogos et al. (2022) showed a 1.85-fold higher prevalence of all types of cancer and a 2.9-fold higher prevalence of breast cancer among female orthopaedic surgeons [8]. The literature does not seem to provide much information on the degree of cancer risk among USs. Chou et al. (2015) revealed in a cross-sectional survey that there was no difference between the observed prevalence and the age, sex, and race-adjusted expected prevalence of breast cancer (both *p* > 0.3) among female urologists. However, it must be noted that the number of all cancer cases (*n* = 7) or breast cancer cases (*n* = 5) in the population (*n* = 328) was very low [6]. 

Furthermore, the eye lens is remarkably radiation-sensitive. The annual dose limit for the lens of the eye was recently reduced by the International Commission on Radiological Protection (ICRP) from 150 Millisievert (mSv) to 20 mSv, since lower than anticipated levels of radiation exposure can lead to cataracts [9,10]. The limit value for organ equivalent doses for the skin and extremities is 500 mSv/a [11]. 

To date, there is scant data on the radiation exposure to which USs are regularly exposed in the endourology operating theatre. Vassileva et al. (2021) demonstrated an eye lens dose ranging from <10 to 63 µSv per surgery in the endourology operating theatre during 315 procedures [12]. Park et al. (2021) conducted a study where the cumulative radiation exposure of the US was measured at 6 locations during 226 RIRS procedures. The following average exposure was recorded: 0.29 mSv for the eye lens, 0.58 mSv for the chest, 0.55–0.73 mSv for the extremities, and 0.31 mSv for the neck [13]. De Coninck et al. (2024) published a systematic review on this topic, which included 21 studies, 14 of which had a prospective design. Some of the studies date back to the 1980s. The highest SCR doses were observed in PCNL, especially in the prone position [14].

In our study, the median radiation exposures were 38.81 µSv for the chest and 17.2 µSv for the head (and thus the eye lens) of the US and 7.71 µSv for the chest and 11.58 µSv for the head of the SN per surgery. Therefore, a US could perform a median of 1162 such surgeries per year without reaching the dose limit for the lens of the eye. However, the maximum eye lens dose per procedure was 73.49 µSv (for RIRS + contralateral (cl) DJ, *n* = 2) in our study. It is important to note that from our viewpoint, the personal exposures of the anaesthetist (** *p* < 0.005) and anaesthesia care (* *p* < 0.05) showed a significant correlation with the DAP, even though the colleagues spend a significant portion of the procedure behind a lead-glass screen. No correlation between exposure and DAP could be demonstrated for the ASN only (chest and head, each *p* > 0.05). As anticipated, the individual SCR doses recorded for the US, SN, A, and AC showed a significant correlation with the corresponding procedure DAP values, highlighting the physical interdependence between the central X-ray beam and the SCR dose. Furthermore, our study shows a significant correlation between the DAP and individual patient factors, including age (** *p* < 0.001), BMI (** *p* < 0.001), and weight (** *p* < 0.001). This finding is consistent with data from Torrecilla Ortiz et al. (2014), who demonstrated a similar correlation between the patient’s BMI and the total radiation dose (*p* < 0.01) [15]. 

In our study, another decisive factor for the DAP was the duration of the surgery (**p < 0.001). Wenzler et al. (2017) also demonstrated a correlation between the individual radiation exposure of staff in the endourology operating theatre during PCNL and factors such as surgery duration (*p* < 0.001) and fluoroscopy time (*p* < 0.001) [16]. The factors discussed above could serve as a predictor for high radiation exposure for the patient and the urology team during procedures, and they call for a heightened awareness to spare the use of fluoroscopy during these procedures. It is worth noting that our research did not detect a correlation between DAP and maximum stone size in RIRS (*p* = 0.10), which is an indication for RIRS (*p* = 0.08), or SFR (*p* = 0.36), while other research groups did demonstrate a correlation between the individual exposure of operating room staff and factors such as stone area (*p* < 0.01) and partial or staghorn calculi (*p* = 0.03) during PCNL [16]. Park et al. (2021) demonstrated that the radiation exposure of the US at the eye lens (*p* < 0.001), right hand (*p* < 0.001), and chest (*p* < 0.001) during SM significantly correlated with the number of stones [13].

Wearing eye lens dosimeters is not a standard practice in every institution. The minimal legal requirement is limited to a thermoluminescence chest dosimeter worn below the lead apron. Previous data from phantom studies and clinical recordings have shown that the eye lens dose can be estimated from the chest dose using a conversion factor. The chest-to-eye conversion factor (CECF) shows a substantial degree of variation based on the underlying data series. A conversion factor with a mean value of 0.48 ± 0.33 for the CECF was recommended by the Swiss Society of Radiobiology and Medical Physics in 2021, based on an analysis of 667 datasets from the Dosilab AG (Gartenstadtstrasse 7A, 3098 Köniz, Switzerland). A literature analysis of 58 peer-reviewed publications by the same working group yielded a CECF ranging from 0.28 to 1.1. Due to the large variability of the results, the working group recommended a CECF of 1 [17]. 

Our research has found a CECF of 2.11 for the US and 0.71 for the SN, highlighting the individual factor differences for different occupational groups. These measures indicate that the US tends to have more exposure in the chest area, while the SNs have more exposure in the head area (i.e., eye lens). This may be related to body height, as most of the USs in our study were men (♂ = 13/♀ = 4), while the majority of SNs were women, who tend to be shorter [18]. Height and, thus, the distance of the eye lens to the radiation source may mean a significant alteration in radiation exposure [19]. Other influencing factors and potential biases with eye dosimeters include head posture and dosimeter position [20,21]. 

Overall, the radiation doses for the SN are lower than those of the US. This may be associated with the greater distance of the SN from the X-ray machine (ALARA principle), but also with the partial shielding of the SN by the US, as the SN moves from right to left behind the US (see Figure 3 for positions).

Various techniques are available to prevent the operating staff from being exposed to radiation. The ALARA principle, i.e., “As Low As Reasonably Achievable”, is a fundamental concept in radiation protection. It underlines the reduction in individuals’ radiation exposure by employing the following methods: time, distance, and shielding [22]. 

Particularly in urology, Tzelves et al. (2023) recommended various measures as part of a review [23]. In the operating room setting, some recommended techniques include the monitoring of the use of fluoroscopy, utilising alarms, employing protective equipment, ensuring the optimal patient positioning, and maintaining maximum distance between staff and the X-ray device. When setting up the X-ray device, attention must be paid to collimation and the use of pulsed fluoroscopy instead of continuous fluoroscopy.

The use of lead aprons, thyroid shields, and lead glasses is of particular importance in urology. Vassileva et al. (2021) indicated an exposure below the regulatory dose limits under lead aprons [12] through an analysis of 6 endourological centres with 44 monitored individuals (US and SN). They also maintained that the use of lead aprons can reduce radiation exposure r by up to 98% in the endourology operating theatre [13]. Wearing lead-glass spectacles can significantly reduce the eye lens dose in urology; e.g., Suzuki et al.’s (2020) findings indicate that this could significantly lower the eye lens dose for two urologists (by 49.2–78.1%) [24]. Nevertheless, they are still not worn regularly.

Mobile lead shields can be used as additional protection even though they were rarely used in the study centre. Our findings reveal that only 5 out of 249 (2.01%) procedures were performed with an additional lead shield. From a personal perspective, some USs find its use impractical as it hinders direct access to the patient (urethra), especially in the lithotomy position. Similarly, no significant reduction in exposure was observed on the dosimeters USC (*p* = 0.479), USH (*p* = 0.127), SNC (*p* = 0.274), or SNH (*p* = 0.203) due to the very small number of data points. Hristova-Popova et al.’s research (2015) used EDD-30 dosimeters and phantoms, and they confirmed that with the application of a 0.35 mm lead shield, the eye lens dose could be reduced from 0.9 mSv/h to 0.06 mSv/h (lithotomy position) and from 1.9 mSv/h to 0.02 mSv/h (PCNL position) [25].

An option for stone removal that completely avoids the use of X-rays is open or laparoscopic lithotomy [26,27]. However, according to the EAU guidelines on urolithiasis, this approach is recommended only in cases where “shock wave lithotripsy, retrograde or antegrade ureteroscopy, and percutaneous nephrolithotomy fail, or are unlikely to be successful” [28]. PCNL access can also be performed safely and effectively using only ultrasound, which would reduce the SCR exposure for both patients and practitioners [29].

In the operating room, a significant element of personal behaviour in dealing with X-rays seems to originate from professional experience. For example, Blattert et al. (2024) claim that junior surgeons in orthopaedic surgery were exposed to significantly higher radiation doses, particularly in the area of the hands (*p* ≤ 0.02), and had longer fluoroscopy times (*p* = 0.019) compared with senior surgeons during the stabilisation of 22 long-bone shaft fractures with intramedullary nails [30]. Unfortunately, our study could not observe a learning curve regarding DAP for one designated resident physician, which may be connected to the relatively short research study period. To address this research question, a larger-scale analysis of DAP data from multiple USs at our clinic would be necessary; thus, it is planned to be carried out in the future.

Radiation protection measures serve not only the patients but also the staff to a great extent. Ongoing research on this important topic would generate data that raise awareness among staff members about the dangers of regular exposure and enable improvements and the implementation of radiation protective measures. This research would be crucial for enhancing safety protocols and minimising health risks. Arslanoglu et al. (2007) concluded that 93% of the surveyed physicians underestimated the patients’ radiation dose through standard X-ray examinations [31]. This finding certainly applies to personal radiation exposure as well. Changes in personnel behaviour can significantly contribute to improvements in patient and staff safety. Murat et al. (2021) maintain that awareness of personal radiation exposure levels in the cardiac catheterisation laboratory leads to a reduction in applied radiation, and thus, exposure [32]. This result can be achieved with the help of real-time dosimeters. James et al. (2015) highlighted that direct feedback through real-time dosimetry during cerebral angiography reduced the surgeon’s exposure by up to 70% and the exposure of the SN by up to 40% [33]. Active training can also lead to a reduction in radiation dose; e.g., Slegers et al. (2015) claimed that interventional pain physicians were able to reduce their radiation exposure by 46% through coaching and active feedback using real-time dosimetry [34].

One of the limitations of our study is the relatively small number of subgroups, such as individual surgeries. Moreover, the professional experience of the USs varies significantly, which can further influence the DAP and therefore the exposure. The dosimeter position on the wearer and his/her position relative to the X-ray device could also denote a potential bias in this study. Further research is needed to accurately assess the individual SCR exposure for each procedure.

## 5. Conclusions 

This study is believed to be one of the first and most comprehensively documented analyses of staff radiation exposure in the endourology operating theatre using real-time dosimetry. The staff was exposed to significant radiation doses, which vary depending on the type of procedure, and they correlate with the DAP. For the US, the exposure in the chest area was higher than in the head area, while the opposite was observed for the SN. Further research would be needed to investigate whether the use of additional lead shields could reduce personal radiation exposure and whether any learning curve may be observed in the application of ionising radiation.

## Figures and Tables

**Table 1:** Characteristics of patients and operative procedures. If not specified otherwise, the mean ± standard deviation (SD) is indicated (cl = contralateral, ESWL = extracorporeal shock wave lithotripsy, PCNL = percutaneous nephrolithotomy, RIRS = retrograde intrarenal surgery, SM = stone management).

		*N* = 249
Demographic patient data	Age (years)	67.23 ± 16.04
	BMI (kg/m^2^)	26.64 ± 6.05
	Body weight (kg)	78.73 ± 18.79
	Height (cm)	172.25 ± 9.11
	*n* male (%)	162 (65.06)
Surgery details	Operating time (minutes)	25.14 ± 26.42
	Surgeries:	
	● *n* RIRS (%)	● 83 (33.33)
	● *n* nephrostomy (%)	● 38 (15.26)
	● *n* MJ ureteral catheter (%)	● 34 (13.65)
	● *n* DJ ureteral catheter (%)	● 73 (29.32)
	● *n* others (%)	● 2 (0.8)
	● *n* PCNL (%)	● 10 (4.02)
	● *n* pyelography (%)	● 3 (1.2)
	● *n* placement of Memokath™ (Memokath™, Oldenvej 13, 3490 Kvistgaard, Denmark; %)	● 1 (0.4)
	● *n* RIRS + cl DJ (%)	● 2 (0.8)
	● *n* nephrostomy + cl DJ (%)	● 2 (0.8)
	● *n* RIRS + ESWL (%)	● 1 (0.4)
	Side of surgery (*n* = 213):	
	*n* left (%)	100 (46.95)
	*n* right (%)	64 (30.05)
	*n* bilateral (%)	49 (23.00)
For RIRS/PCNL	Maximum stone size in mm (*n* = 55)	10.52 ± 9.13
	If RIRS (*n* = 84):	
	indication for SM *n* (%)	48 (57.14)
	diagnostic *n* (%)	36 (42.86)
	Use of laser yes *n* (%) (*n* = 61)	35 (57.38)
	SFS positive (%) (*n* = 60)	49 (81.67)
	Use of anticoagulants/antiplatelet therapy yes (*n* = 87, %)	23 (26.44)
	Urinary stone analysis (*n* = 56)	
	● *n* Calcium oxalate (%)	● 35 (62.50)
	● *n* Carbonated apatite (%)	● 12 (21.43)
	● *n* Uric acid (%)	● 8 (14.29)
	● *n* Brushite (%)	● 1 (1.79)
	Stone localisation (*n* = 59):	
	*n* ureter (%)	17 (28.81)
	*n* renal pelvis (%)	35 (59.32)
	*n* both (%)	7 (11.86)

**Table 2:** Median values of individual dosimeters according to type of surgery (50% confidence interval (CI)); AC = anaesthetist chest, ACC = anaesthesia care chest, ASNC/H = assistant surgical nurse chest/head, cl = contralateral, ESWL = extracorporeal shock wave lithotripsy, PCNL = percutaneous nephrolithotomy, RIRS = retrograde intrarenal surgery, SNC/H = surgical nurse chest/head, Sv = Sievert, USC/H = urological surgeon chest/head. Comment: Measurement for USH/Memokath™ removed due to implausibly low recording).

Type of Surgery	Radiation Exposure in Microsievert (µSv)							
	USC	USH	SNC	SNH	ASNC	ASNH	AC	ACC
RIRS (83)	37.65 (16.92–55.53)	22.17 (8.93–48.75)	8.04 (3.56–15.20)	11.45 (4.66–22.90)	0.08 (0.03–0.13)	0.10 (0.04–0.29)	0.43 (0.12–1.22)	0.18 (0.08–0.36)
Nephrostomy (38)	29.20 (8.60–60.94)	5.94 (2.03–31.84)	7.38 (4.62–17.65)	9.37 (5.61–25.94)	0.05 (0.03–0.15)	0.05 (0.03–0.33)	5.45 (0.41–8.14)	
MJ ureteral catheter (34)	47.62 (31.04–97.17)	23.26 (8.15–42.88)	7.26 (4.75–17.07)	8.80 (4.92–25.17)	0.46 (0.33–0.69)	1.11 (0.28–1.87)	5.18 (0.30–10.06)	
DJ ureteral catheter (73)	41.01 (17.38–99.36)	17.21 (6.07–39.37)	7.16 (4.26–18.42)	13.85 (6.06–33.88)	0.17 (0.08–0.99)	0.33 (0.05–0.56)	1.36 (1.09–1.71)	0.32 (0.09–0.93)
Others (2)	39.14 (39.14–39.14)	2.02 (0.03–4.01)						
PCNL (10)	47.44 (16.18–248.89)	19.62 (15.96–57.70)	19.30 (9.77–59.59)	27.68 (11.71–97.63)	0.44 (0.38–1.09)	0.39 (0.33–0.68)	5.19 (3.53–6.84)	2.28 (0.26–4.30)
Pyelography (3)	2.57 (0.97–4.17)	3.44 (3.44–3.44)	6.20 (2.92–35.40)	11.89 (4.12–24.56)	0.39 (0.05–0.72)	0.35 (0.03–0.66)		
Memokath™ insertion (1)			154.45 (154.45–154.45)	183.44 (183.44–183.44)	0.59 (0.59–0.59)	0.52 (0.52–0.52)		
RIRS + cl DJ (2)	149.96 (104.13–195.79)	73.49 (37.33–109.65)						
Nephrostomy + cl DJ (2)	54.30 (54.30–54.30)	21.80 (21.80–21.80)	20.58 (10.24–30.92)	32.32 (17.15–47.48)	40.94 (40.94–40.94)	45.44 (45.44–45.44)		0.13 (0.13–0.13)
RIRS + ESWL (1)	149.07 (149.07–149.07)			0.23 (0.23–0.23)	0.18 (0.18–0.18)	0.18 (0.18–0.18)	0.26 (0.26–0.26)	0.18 (0.18–0–18)
Total (249)	38.81 (17.89–80.53)	17.20 (5.46–39.57)	7.71 (4.18–17.81)	11.58 (5.35–27.02)	0.12 (0.05–0.51)	0,25 (0.05–0.54)	0.63 (0.20–2.22)	0.23 (0.10–0.54)

**Table 3:** Median chest-to-eye conversion factor (CECF) of urological surgeon and surgical nurse (CI = confidence interval).

	*n*	Median CECF (50% CI)
Urological surgeon	152	2.11 (1.97–2.28)
Surgical nurse	201	0.71 (0.64–0.75)

## Data Availability

The data presented in this study are available on request from the corresponding author due to ethical reasons.

## Supplementary Materials

The following supporting information can be downloaded at: https://www.mdpi.com/article/10.3390/diagnostics14161763/s1, Table S1: median dose area product (DAP) broken down by type of surgery (50% confidence interval (CI), cGy = centigray). Table S2: number of surgeries performed according to the professional experience and position of the urological surgeon (US) (ESWL = Extracorporeal Shock Wave Lithotripsy, PCNL = percutaneous nephrolithotomy, RIRS = retrograde intrarenal surgery).

## Abbreviations

A, AC	Anaesthetist, Anaesthetist chest
AC, ACC	Anaesthesia care, Anaesthesia care chest
cGy	Centigray
cl	Contralateral
CSLS	Ceiling-supported lead shield (CSLS)
DAP	Dose area product
ESWL	Extracorporeal shock wave lithotripsy
OSCAR	Occupational SCAtter Radiation Registry
PCNL	Percutaneous nephrolithotomy
RIRS	Retrograde intrarenal surgery
SFS	Stone-free status
SM	Stone management
SN, SNC/H	Surgical nurse, surgical nurse chest/head
Sv	Sievert
m/µSv	Milli-/Microsievert
US, USC/H	Urological surgeon, urological surgeon chest/head
