# Supplementary material for: Real-Time Dosimetry in Endourology: Tracking Staff Radiation Risks
Source: Diagnostics (Basel). 2024 Aug 13;14(16):1763. doi: 10.3390/diagnostics14161763 (PMC11353960; doi:10.3390/diagnostics14161763)
Supplement: Supplementary file 1 [file diagnostics-14-01763-s001.zip › diagnostics-3127928-supplementary.pdf]

Supplementary material:

Surgeries (n)	DAP in cGy·cm^2
**RIRS (83)**	391.20 (234.20 – 598.30)
**nephrostomy (38)**	136.20 (89.10 – 370.30)
**MJ ureteral catheter (34)**	277.40 (153.10 – 367.50)
**DJ ureteral catheter (73)**	447.20 (196.90 – 742.90)
**Others (2)**	55.50 (1.60 – 109.40)
**PCNL (10)**	716.85 (454.30 – 2131.30)
**Pyelography (3)**	184.60 (66.70 – 404.10)
**Memokath™ insertion (1)**	3231.30 (3231.30 – 3231.30)
**RIRS + DJ ureteral catheter (2)**	898.80 (599.10 – 1198.50)
**nephrostomy + DJ ureteral catheter (2)**	690.40 (253.70 – 1127.10)
**RIRS + ESWL (1)**	801.00 (801.00 – 801.00)
**Total (249)**	328.10 (1.60 – 606.80)

Table S1: *median dose area product (DAP) broken down by type of surgery (50% confidence interval (CI), cGy=centigray).*

Surgery	n total	Professional experience/ Position of US		
		resident physician<br>in %	Urology specialist<br>in %	Urology expert<br>in %
**RIRS**	83	10.00	51.95	45.83
**nephrostomy**		21.00	7.79	15.28
**MJ ureteral catheter**	34	24.00	5.19	8.33
**DJ ureteral catheter**	73	45.00	16.88	20.83
**others**	2	0	1.30	1.39
**PCNL**	10	0	10.39	2.78
**pyelography**	3	0	1.30	2.78
**Memokath™ insertion**	1	0	0	1.39
**RIRS + DJ ureteral catheter**	2	0	2.60	0.00
**nephrostomy + DJ ureteral catheter**	2	0	1.30	1.39
**RIRS + ESWL**	1	0	1.30	0.00
**Total**	249	100.00	100.00	100.00

Table S2: *number of surgeries performed according to the professional experience and position of the urological surgeon (US) (ESWL= Extracorporeal Shock Wave Lithotripsy, PCNL= percutaneous nephrolithotomy, RIRS= retrograde intrarenal surgery).*
